# Telomerase: A Target for Therapeutic Effects of Curcumin and a Curcumin Derivative in Aβ1-42 Insult *In Vitro*

**DOI:** 10.1371/journal.pone.0101251

**Published:** 2014-07-01

**Authors:** Zijian Xiao, Aiwu Zhang, Jianwen Lin, Zhenyang Zheng, Xiaolei Shi, Wei Di, Weiwei Qi, Yingting Zhu, Guijuan Zhou, Yannan Fang

**Affiliations:** 1 Department of Neurology, the First Affiliated Hospital, Sun Yat-sen University, Guangzhou, China; 2 Zhongshan Ophthalmic Center, Sun Yat-sen University, Guangzhou, China; University of Newcastle, United Kingdom

## Abstract

This study was designed to investigate whether telomerase was involved in the neuroprotective effect of curcumin and Cur1. Alzheimer's disease is a consequence of an imbalance between the generation and clearance of amyloid-beta peptide in the brain. In this study, we used Aβ1-42 (10 µg/ml) to establish a damaged cell model, and curcumin and Cur1 were used in treatment groups. We measured cell survival and cell growth, intracellular oxidative stress and hTERT expression. After RNA interference, the effects of curcumin and Cur1 on cells were verified. Exposure to Aβ1–42 resulted in significant oxidative stress and cell toxicity, and the expression of hTERT was significantly decreased. Curcumin and Cur1 both protected SK-N-SH cells from Aβ1–42 and up-regulated the expression of hTERT. Furthermore, Cur1 demonstrated stronger protective effects than curcumin. However, when telomerase was inhibited by TERT siRNA, the neuroprotection by curcumin and Cur1 were ceased. Our study indicated that the neuroprotective effects of curcumin and Cur1 depend on telomerase, and thus telomerase may be a target for therapeutic effects of curcumin and Cur1.

## Introduction

Although the exact etiology of Alzheimer's disease (AD) has not been elucidated, the most widely accepted theory is the “amyloid cascade theory”, which proposes that AD is a consequence of an imbalance between the generation and clearance of amyloid-beta (Aβ) peptide in the brain [1]. On a molecular basis, cell cycle changes and oxidative stress resulting from increase of reactive oxygen species (ROS) have also been shown to play detrimental roles in AD [2]. The Aβ-induced oxidative stress hypothesis [3], [4] states that Aβ and the damage it initiates are the underlying mechanism leading to injurious increase of oxidative stress in AD brain.

**Figure 1:**
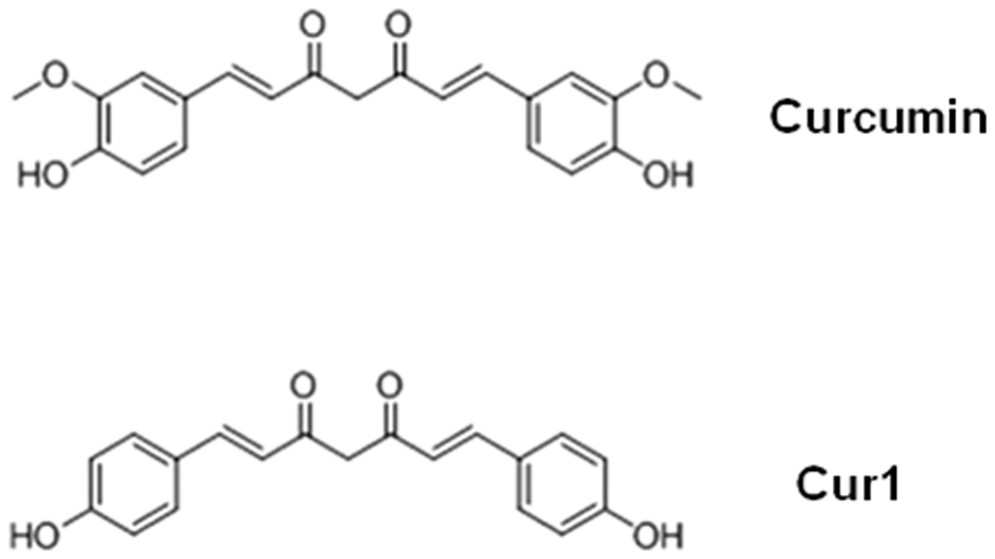
Chemical structure formula of curcumin and Cur1.

Curcumin is a polyphenolic compound abundant in the Indian spice turmeric. Animal studies have confirmed neuroprotective effects of the drug in neurodegenerative conditions such as AD [5], [6], [7]. In particular, curcumin could reduce oxidative damage and alleviate amyloid pathology in AD [7], [8], [9]. In vivo studies showed that curcumin could promote disaggregation of existing amyloid deposits, prevent aggregation of new amyloid deposits, and reduce amyloid deposits [10]. In our previous study [11], among curcumin and its six derivatives, curcumin and Cur1 (the 3-methoxy at both ends of benzene ring was removed, but 4-hydroxyl retained) (Fig. 1) showed neuroprotective effects.

Telomeres, highly conserved repetitive DNA sequences, undergo shortening not only with each cell division [12], [13], but also with aging and oxidative stress—all involved in AD. Telomere length reflects cellular replicative history. Once telomere reaches a critical minimum length, the cells become senescent [12]. However, at the same time dividing cells express telomerase, a ribonuclear protein complex that synthesizes and elongates telomeric DNA [14]. Human telomerase reverse transcriptase (hTERT), the catalytic subunit of telomerase, functions to stabilize telomere length during chromosomal replication [15].

Thus, we hypothesise that the expression of hTERT could be enhanced by curcumin and that telomerase is a target for the therapeutic effects of curcumin against Aβ insult. However, some studies on cancer [16], [17] found that curcumin induced cell death through inhibition of telomerase activity in human cancer cells. And the present study was designed to further investigate whether telomerase was involved in the neuroprotective effect of curcumin and Cur1.

## Materials and Methods

Human SK-N SH cells from Experimental Animal Center of Sun Yat-sen University were used in this study. Curcumin, Cur1, trypsin, MTT, DMSO and Aβ 1–42 were purchased from Sigma-Aldrich. Culture media and FBS were purchased from Gibco. A rabbit monoclonal anti-hTERT antibody was purchased from Millipore. Rhodamine goat anti- rabbit IgG were purchased from Chemicon. HRP goat anti-rabbit IgG and anti-β-actin were purchased from Boshide. Bisbenzimide trihydrochloride Hoechst 33258 was from Alexis. Reactive Oxygen Species Assay Kit was from Beyotime. GSH Assay Kit was from Keygen BioTECH. The siRNAs and RNase-free water were from GenePharma. Lipofectamine™ 2000 and primers were from Invitrogen. RNAiso Plus, PrimeScript RT Enzyme Mix I and SYBR Premix Ex Taq II kits were from TaKaRa.

### Cell preparation

Control group: SK-N-SH cells were cultured in normal medium (DMEM/F12 containing 10% FBS). Injury group: The use of Aβ1-42 peptides was based upon previous studies, because Aβ1-42 can induce the neuritic and synaptic toxicity, and aggregates more easily and forms more plaques than Aβ1-40. We chose Aβ1-42 (10 µg/ml) to establish our damaged cell model, because other concentrations could cause serious injury to cells or too minor injury to be easily observed [11]. Added 10 µg/ml Aβ1-42 to SK-N-SH cells and incubated them for 24 h, and the cells were subsequently cultured for 3 days in normal medium. Treatment groups: Added 10 µg/ml curcumin and Cur1 respectively before addition of Aβ1-42 (10 µg/ml),and incubated them for 24 h.

### Quantification of viability by MTT

Cell survival and cell growth was tested by MTT assay. MTT assay was carried out as follows, 20 µl MTT (5 mg/ml) added to cells and incubated for 4 h in the groups mentioned above, then 150 µl DMSO was added to every well and fluorescence measured with an excitation wavelength at 490 nm and an emission wavelength at 570 nm.

### Detection of reactive oxygen species with fluorescent probe

Intracellular reactive oxygen species (ROS) was analyzed using 2′, 7′-dichlorodihydrofluorescein diacetate (DCFH-DA) as a probe. DCFH-DA enters cells where intracellular esterases cleave off the diacetate group. The resulting DCFH is retained in the cytoplasm and is oxidized to 2′, 7′-dichlorofluorescein (DCF) by ROS. SK-N-SH cells were treated with the designated experimental conditions and were collected and incubated with DCFH-DA for 30 min at 37°C. Then the cells were washed three times with phosphate buffered saline (PBS, pH = 7.4) and the intracellular accumulation of fluorescent DCF were measured with excitation 488 nm and emission 525 nm, respectively. The results were expressed as intensity of DCF-fluorescence and compared with controls.

### Determination of glutathione (GSH)

GSH level was used to indicate the intracellular oxidative stress. The content of GSH was measured using a GSH Assay Kit (Keygen BioTECH, Nanjing, China). The GSH content was determined by reacting with 5, 5′-dithiobis-2-itrobenzoic acid (DTNB) to form a yellow-colored product, which can be measured by colorimetry. Briefly, SK-NSH cells were seeded in six-well plates (1×10^6^ cells/well) and treated with the designated experimental conditions. The cells were collected by 0.25% trypsin (w/v)/0.02% EDTA (w/v) and lysed in lysis buffer [0.05 mM EDTA, 1% Triton-X 100 (v/v), pH = 8.0] for 1 h at 4°C, and then the cell suspension was centrifuged at 12,000 rpm for 2 min. Subsequently, 1.9 ml freshly prepared disodium hydrogen phosphate buffer and 0.5 ml of 0.004% DTNB (w/v) was added into the supernatant. The optical density of the yellow-colored complex formed by the reaction of GSH and DTNB was measured by a spectrometer at a wavelength of 420 nm. The GSH content was calculated from a standard curve of GSH slope, and normalized to the protein concentration, which was determined by Coomassie Brilliant Blue method.

### Immunocytochemical staining

Cells were fixed with formaldehyde (4% paraformaldehyde, freshly depolymerized, in 0.1 M PBS, pH = 7.4) for 15 min at room temperature and washed three times in PBS. Blocked with blocking buffer [5% BSA (w/v), 0.1% Triton X-100 in PBS (w/v), pH = 7.4] for 1 h at room temperature. After blocking, cells were incubated with the primary antibody (anti- hTERT 1∶100) in dilute solution [2% BSA, 0.3% Triton X-100 in PBS, pH = 7.4] at 4°C overnight, and then the cells were washed and incubated with the secondary antibody conjugated to Rhodamine for 2 h at room temperature. The cells were washed and then stained with Hoechst 33258 nuclear for 15 min at room temperature. Finally, the immunofluorescence staining of hTERT was observed under inverted phase contrast microscope (BX51, Olympus, Japan).

### Western blotting analysis

Samples were taken from whole cell culture in groups, and then the proteins of 10 µl sample were separated using gel electrophoresis and transferred to polyvinylidene difluoride (PVDF) membranes. PVDF membranes were blocked 2 hours at room temperature in TBST containing 5% BSA. Subsequently, the membranes were incubated with hTERT monoclonal antibody (material information, 1∶1000) or β-actin monoclonal antibody (material information, 1∶1000) at 4°C overnight. Specific protein expression was then detected by incubating the washed membranes with horseradish peroxidase (HRP) conjugated secondary antibodies (material information, 1∶1000) at room temperature for 1 h. The fluorescence labels allowed the placement of X-ray film against nitrocellulose membrane as it was exposed to the labels and created dark regions which correspond to the protein bands of hTERT or β-actin.

### Total RNA isolation and quantitative real-time PCR analysis

For total RNA isolation from SK-N-SH cells, RNAiso Plus was added to the six-well plates. After incubating for 5 min at room temperature, chloroform was added for phase separation. The upper aqueous phase was collected and the RNA was precipitated by mixing with isopropyl alcohol, and then the RNA pellet was washed once with 75% ethanol and was air-dried for 5 minutes. It was finally redissolved in small amounts of RNase-free water. The absorbance of the RNA solution was determined using UV spectrophotometer at 260 and 280 nm, respectively.

The first strand cDNA was synthesized from 1 µg of total RNA by Reverse Transcriptase using PrimeScript RT Enzyme Mix I and oligo (dT) primers according to the manufacturer's protocol. Real-time quantitative PCR (RT-PCRq) was performed by SYBR Premix Ex Taq II kit. 2 µl template cDNA were added to the final volume of 20 µl of reaction mixture. Real-time PCR cycle parameters included 30 s at 95°C followed by 40 cycles involving denaturation at 95°C for 5 s, annealing at 60°C for 34 s and elongation at 72°C for 20 s. The following primers were used: for hTERT, forward: 5′-TGC TGA CGT CCA GAC TCC G-3′; reverse: 5′-TCT GGC TCC CAC GAC GTA GT-3′; for β-actin, forward: 5′-GGAGATTACTGCCCTGGCTCCTA-3′; reverse: 5′-GACTCATCGTACTCCTGCTTGCTG-3′. Expressions of hTERT were corrected to β-actin gene, which was used as an internal housekeeping control. For relative quantification of hTERT, we used the comparative CT method (ΔΔCT). All the real-time PCR experiments were performed in triplicate and data expressed as the mean of at least five independent experiments.

### Transient Transfection with Small Interfering RNAs (SiRNAs)

Three siRNAs, designed to knock down expression of hTERT, were purchased from GenePharma (GenePharma, Suzhou, China). TERT-homo-1797: GGAGCAAGUUGCAAAGCAUTT, AUGCUUUGCAACUUGCUCCTT; TERT-homo-2919: GAGCCAGUCUCACCUUCAATT, UUGAAGGUGAGACUGGCUCTT; TERT-homo-3042: CGGUGUGCACCAACAUCUATT, UAGAUGUUGGUGCACACCGTT. In brief, SK-N-SH cells were cultured to 60–80% confluence in complete DMEM for three days at 37°C in a CO_2_ incubator before transfection. The siRNA transfection reagent mixture diluted in DMEM without FBS was then added to cells that had been washed twice with PBS. After 6 h, the medium was replaced with complete medium. Cells were then incubated for further 24 h before injury and treatments as mentioned above. Control siRNAs were used as a negative control. Transfection reagent control group: We added lipofectamine 2000 into SK-N-SH cells to investigate whether lipofectamine 2000 had toxic effect on cells. The efficiency of transfection was verified by quantitative real-time PCR and western blot.

### Statistical analysis

Statistical analyses were performed using SPSS 16.0 and the results were expressed as the mean ± SD. Differences between the means were determined by one-way ANOVA and t-test was used between each two groups. In all statistical analyses, *P*<0.05 was regarded as statistically significant.

## Results

### Protective effect of curcumin and Cur1

#### 1. Improving cell viability

**Figure 2:**
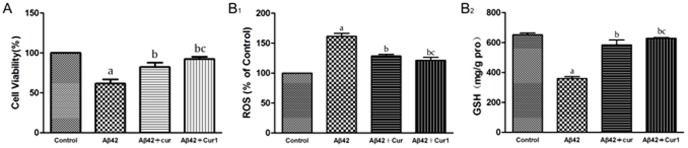
A: MTT assay of cell viability after treatment with curcumin and Cur1. B1: Effects of curcumin and Cur1 on generation of intracellular ROS. After damage by Aβ1-42, intracellular ROS increased as determined by evaluation of DCF fluorescence. The increase in ROS was inhibited by the presence of curcumin and Cur1. B2: Effects of curcumin and Cur1 on GSH, a sensitive indicator of oxidative stress. Curcumin and Cur1 both increased the expression of GSH in SK-N-SH cells. Results are represented as mean ± SD from five independent experiments. a: P<0.001 vs. control group. b: P<0.001 vs. Aβ1-42 group. c: P<0.01 vs. curcumin group.

Viability of SK-N-SH cells was reduced to approximately 62% of baseline after a 24 h exposure to Aβ1–42 (*P*<0.001) and curcumin and Cur1 protected against reducing in cell viability (Fig. 2A). There was statistical difference between the curcumin group and the Cur1 group were compared to the Aβ1–42 group (*P*<0.001). Curcumin and Cur1 could prevent cells from the cytotoxicity of Aβ1–42 as shown by improved cell viability, while Cur1 showed a more significant protective effect (92%) than curcumin (82%, *P*<0.05). These results suggested that both of curcumin and Cur1 could improve cell viability, particularly Cur1.

#### 2. Antioxidant effects

As shown in Figure 2B, ROS was increased significantly (*P*<0.001) and GSH was declined significantly (*P*<0.001) after exposure to Aβ1–42 for 24 h. However, there was a significant decrease in ROS generation and a significant increase in GSH level (p<0.001) when pretreated with curcumin or Cur1 (*P*<0.001). In addition, Cur1 exhibited better antagonistic effect on Aβ1–42-induced oxidative stress than curcumin (*P*<0.05). These findings indicate that both curcumin and Cur1 could reduce oxidative stress.

#### 3. Up-regulation of hTERT

**Figure 3:**
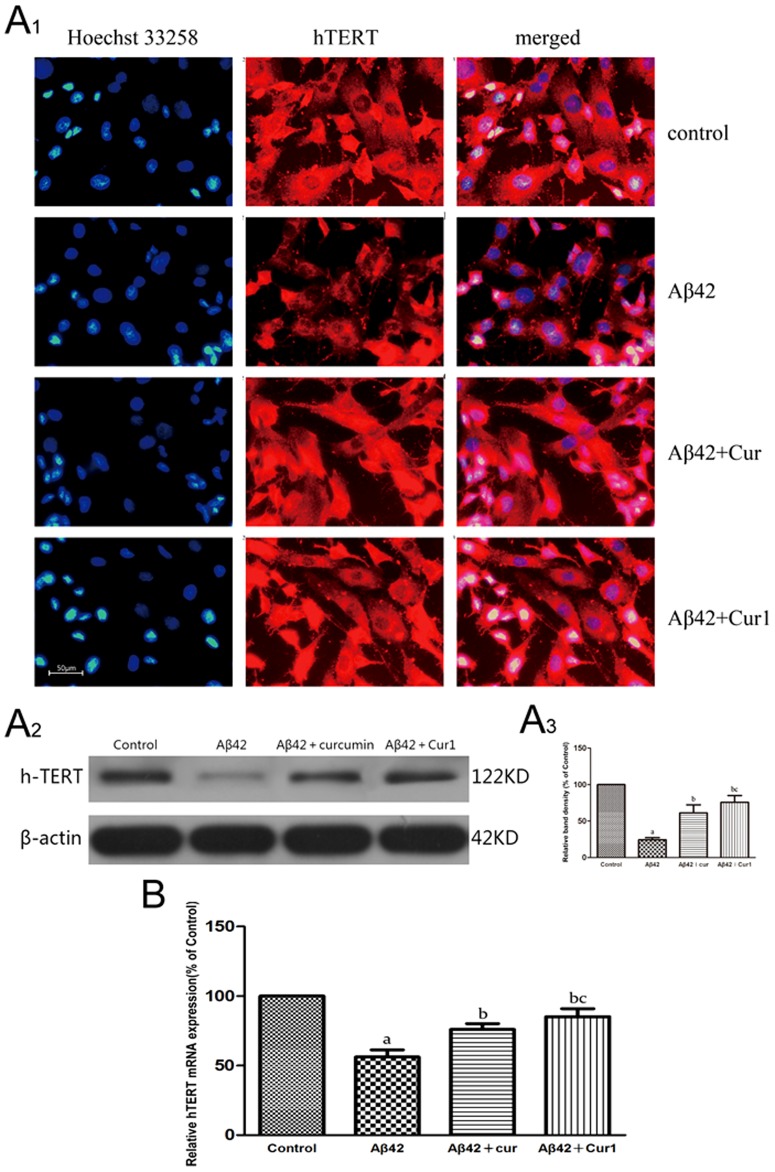
Effects of curcumin and Cur1 on hTERT. A: analysis of hTERT protein expression by fluorescent immunocytochemistry(A1) and western blot(A2). A3: Quantitative analysis of western blot (A2). B: analysis of hTERT mRNA expression by real-time PCR. After addition of Aβ1-42, expression of hTERT at protein and mRNA level both declined, which were reversed in the presence of curcumin and Cur1. Curcumin and Cur1 both increased the expression of hTERT in SK-N-SH cells. Results are represented as mean ± SD from five independent experiments. a: P<0.001 vs. control group. b: P<0.001 vs. Aβ1-42 group. c: P<0.05 vs. curcumin group.

After incubation of SK-N-SH cells with Aβ1–42, both mRNA and protein level of hTERT declined (*P*<0.001), and hTERT expression was partially restored by curcumin and Cur1 (*P*<0.001) (Fig.3). In addition, Cur1 exhibited a more potent effect of hTERT expression at mRNA and protein level (*P*<0.05). We propose that Aβ1–42 significantly inhibit the expression of hTERT, and curcumin and Cur1 could up-regulate hTERT, both restoring mRNA and protein levels. Plenty of SK-N-SH cells were apoptotic after exposure to Aβ1–42 for 24 h, and some cells with a little cytoplasm were observed. Subsequent treatment with curcumin and Cur1, the numbers of cells were increased (Fig. 3A_1_).

**Figure 4:**
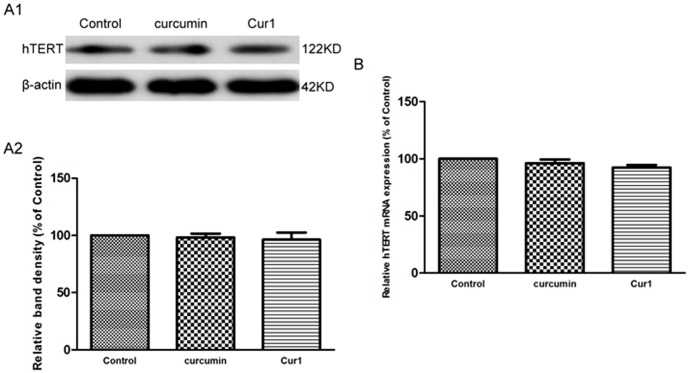
Effects of curcumin and Cur1 on hTERT in absence of Aβ1–42. A1: analysis of hTERT protein expression by western blot. A2: Quantitative analysis of western blot. B: analysis of hTERT mRNA expression by real-time PCR. In absence of Aβ1-42, neither curcumin nor Cur1 increased the expression of hTERT in SK-N-SH cells. There was no significant difference among the three groups. Results are represented as mean ± SD from five independent experiments.

However, neither protein nor mRNA level of hTERT increased when curcumin or Cur1 treated SK-N-SH cells without Aβ1–42 treatment, and there were no significant difference comparing the study groups (*P*>0.05, Fig.4). We propose that curcumin and Cur1 have no effect on hTERT up-regulation in normal cells.

### Down-regulation of hTERT by siRNA

#### 1. Transfection rate

**Figure 5:**
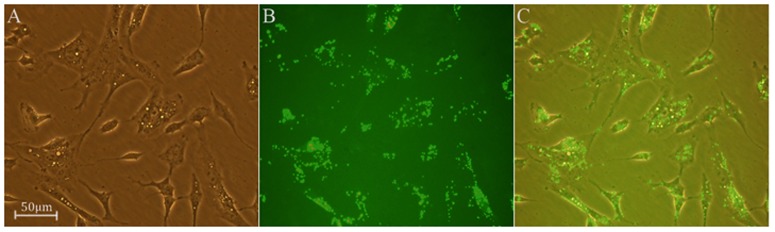
SK-N-SH cells were observed and transfection efficiency was evaluated under fluorescent microscopy. A: cells were observed by ordinary light. B: siRNAs with FAM fluorescence. C: merged picture. The transfection efficiency was over 95%.

As shown in Figure 5, the transfection efficiency of siRNAs into SK-N-SH cells was over 95%.

#### 2. Discovery of the most efficient siRNA

**Figure 6:**
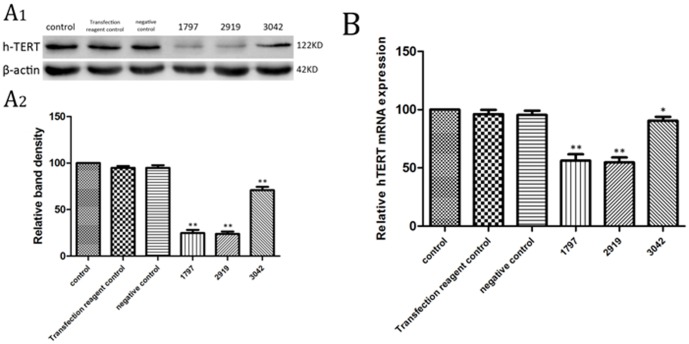
Effects of siRNAs on hTERT. A1: analysis of hTERT protein expression by western blot. A2: Quantitative analysis of western blot (A1). B: analysis of hTERT mRNA expression by real-time PCR. The hTERT at both the protein level and the mRNA level were reduced by all three siRNAs, and 2919 being the most potent in reducing hTERT expression. Results are represented as mean ± SD from five independent experiments. *: P<0.01 vs. control group. **: P<0.001 vs. control group.

We found that hTERT expression could be reduced by all three siRNAs tested and that 2919 was the most potent of them (Fig.6). In present study, TERT-homo-2919 (GAGCCAGUCUCACCUUCAATT, UUGAAGGUGAGACUGGCUCTT) transfected SK-N-SH cells served as an hTERT knock-down group for subsequent experiments.

#### 3. Irreversible inhibition of hTERT by siRNA

**Figure 7:**
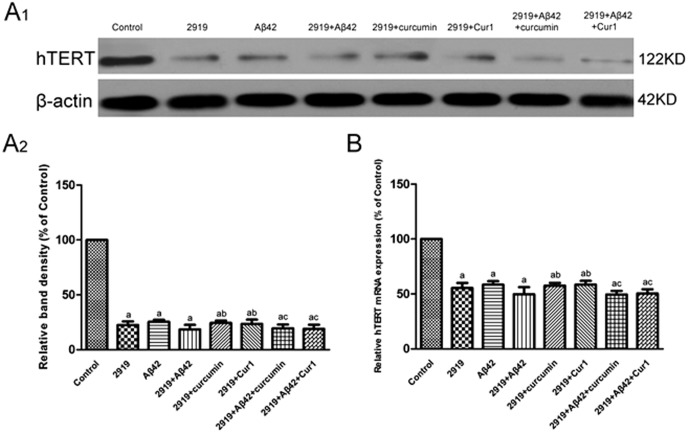
Effects of siRNAs on hTERT. A1: analysis of hTERT protein expression by western blot. A2: Quantitative analysis of western blot (A1). B: analysis of hTERT mRNA expression by real-time PCR. The hTERT at both the protein level and the mRNA level declined after a 24-h exposure to Aβ1–42, RNA interference or both of the two conditions. Curcumin and Cur1 no longer up-regulated the expression of hTERT while there was hTERT knock-down. Results are represented as mean ± SD from five independent experiments. a: P<0.01 vs. control group. b: P>0.05 vs. 2919 group. c: P > 0.05 vs. 2919+ Aβ42 group.

Both mRNA and protein level of hTERT declined after a 24-h exposure to Aβ1–42 (*P*<0.001) or after RNA interference (*P*<0.001) respectively. Furthermore, the expression of hTERT was also reduced after both of the two conditions (*P*<0.001) (Fig. 7). However, curcumin and Cur1 no longer up-regulated the expression of hTERT while there was hTERT knock-down. These results suggested that the ability of curcumin or Cur1 to increase telomerase expression vanished and the inhibition of hTERT by TERT-homo-2919 is irreversible.

### Suppression of hTERT negates protective effects of curcumin and Cur1

#### 1. Knock-down of hTERT negates the effects of curcumin and Cur1 on improving cell viability

**Figure 8:**
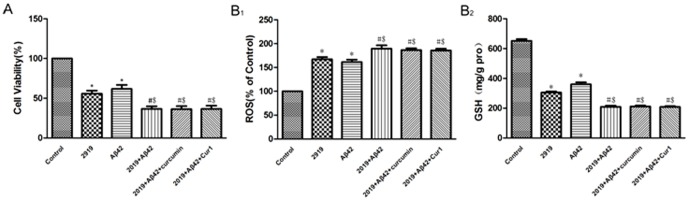
A: MTT assay of cell viability after RNA interference with treatment of curcumin and Cur1. B1: Effects of curcumin and Cur1 on generation of intracellular ROS after RNA interference. B2: Effects of curcumin and Cur1 on GSH after RNA interference. Following knock-down of hTERT, curcumin and Cur1 both could no longer increase the expression of GSH. Results are represented as mean ± SD from five independent experiments. *: P<0.001 vs. control group. #: P<0.001 vs. 2919 group. $: P<0.001 vs. Aβ42 group. There is no statistical difference between the 2919+Aβ42 group, 2919+Aβ42+curcumin group and 2919+Aβ42+Cur1 group (P>0.05).

The viability of SK-N-SH cells was reduced to approximately 62% or 56% of baseline after a 24-h exposure to Aβ1–42 (*P*<0.001) or after RNA interference (*P*<0.001) respectively. Furthermore, the cells viability was reduced to approximately 37% after both of the two conditions (*P*<0.001) (Fig. 8A). However, curcumin (36.26%) and Cur1 (36.60%) no longer showed protective effects on cells while there was hTERT knock-down. There was no statistical difference between the 2919+Aβ42 group, 2919+Aβ42+curcumin group and 2919+Aβ42+Cur1 group (*P*>0.05). These results suggested that both curcumin and Cur1 no longer able to improve cell viability following RNA interference.

#### 2. Knock-down of hTERT negates the antioxidant actions of curcumin and Cur1

As shown in Figure 8B, ROS was significantly increased (*P*<0.001) and GSH was decreased significantly (*P*<0.001) after exposure to Aβ1–42 for 24 h, or after RNA interference. However, curcumin and Cur1 no longer showed the antioxidant effects on cells following RNA interference. There was no statistical difference among 2919+Aβ42 group, 2919+Aβ42+curcumin group and 2919+Aβ42+Cur1 group (*P*>0.05). These findings indicate that both of curcumin and Cur1 lost their ability to reduce oxidative stress after RNA interference.

## Discussion

In this study, SK-N-SH cells were used as an in vitro model of AD. SK-N-SH is a human derived neuroblastoma cell line, and these cells are often used as in vitro models of neuronal function and differentiation because they can simulate the biological characteristics of neurons preferably. However, there are some differences between SK-N-SH cells and neurons. The main difference is that SK-N-SH cells have obvious traits of tumor cells and their axons and dendrites are very different from the true neuronal neuritis.

At present, the mechanisms underlying AD remain unknown, and a well-recognized optimal prevention or therapy for the disorder is still lacking [18]. It has been proposed that Aβ, a major component of senile plaques in the brain, may be involved in neuronal death. High correlations between oxidative stress and cognitive impairment suggest an important role of oxidative stress in the development of AD [19]. Oxidative stress originates from the imbalance between prooxidants and antioxidants which leads to accumulation of ROS [20]. These reactive species can provoke cell membrane lipid destruction, DNA cleavage, oxidation of proteins, and finally apoptosis [21]. In our study, 24-h exposure to Aβ1–42 resulted in significant oxidative stress and cell toxicity in SK-N-SH cells.

Telomerase is an enzyme that adds DNA sequence repeats to the 3′ end of DNA strands in the telomere regions, located at the ends of eukaryotic chromosomes. Human telomerase consists of three molecules each of human telomerase reverse transcriptase (TERT), telomerase RNA (TR or TERC), and dyskerin (DKC1) [22]. TERT can create single-stranded DNA using a template of single-stranded RNA. Short telomeres have been found in some premature aging syndromes [23], and studies have indicated the potential of a therapeutic strategy based on telomerase as an anti-aging target. Accordingly, one study [24] showed that those long-lived individuals inherited a hyperactive telomerase which could rebuild telomeres, and another study reported that introducing the TERT gene into healthy one-year-old mice led to a 24% increase in lifespan [25]. Recently study showed that increasing TERT expression by novel compound confers resistance from apoptosis induced by oxidative stress [26]. Previous studies [27] have suggested that there were a protected mtDNA, a significantly elevated mitochodria membrane potential and a significantly reduced oxidative stress level in hTERT over-expression cells. And the suppressed telomerase activity caused oxidative stress increase and mitochondria dysfunction in embryonic rat hippocampal neurons [28].

Zhu H et al. [29] first reported that suppression of TERT in embryonic mouse hippocampal neurons using antisense technology and the telomerase inhibitor 3′ -azido-2′ 3′ -dideoxythymidine significantly increases neurons vulnerability to cell death induced by Aβ. Neurons in which TERT levels were reduced exhibited increased levels of oxidative stress and mitochondrial dysfunction following exposure to Aβ. Over expression of TERT in pheochromocytoma cells resulted in decreased vulnerability to Aβ-induced apoptosis. One previous study also found that low doses of Aβ could significantly inhibit telomerase activity in vitro [30], and the real-time PCR and western blot analysis in our study further documented that the expression of hTERT was decreased significantly in SK-N-SH cells upon exposure to Aβ1–42. Notably, the reduction in cell viability was simultaneously associated with decreased hTERT expression. We hypothesise that altered cellular proliferation due to Aβ are associated with the inhibition of hTERT.

Further experiments were necessary to clarify whether curcumin and Cur1 could enhance cell viability and restore to normal levels of hTERT following Aβ-induced damage. Abundant reports have demonstrated that curcumin could promote disaggregation of existing amyloid deposits and prevent aggregation of new amyloid deposits [31], straighten the neurites with abnormal curvature near or even far from the senile plaque, and reverse neuritic abnormalities [32]. In our study, curcumin and Cur1 improved cell viability obviously, and we found that both protected SK-N-SH cells from Aβ1–42. Furthermore, increased ROS and reduced GSH levels in the cells due to Aβ1–42 were also significantly prevented by preincubation with 10 µg/ml curcumin and Cur1. These data, therefore, strongly supported the protective effects of curcumin and Cur1 against cellular oxidative stress. Previous studies [7], [8], [9] also suggested that curcumin had particular antioxidant activity and could alleviated amyloidosis. Curcumin could increase the levels of anti-inflammatory cytokine i.e. IL-10 and antioxidant enzymes i.e. superoxide dismutase, catalase and glutathione peroxidase [33], and oxygenase-1 and GSH play a major role in the protective effect of curcumin [34]. Finally, the expressions of hTERT were up-regulated by curcumin and Cur1 against Aβ-induced damage. While hTERT was inhibited by Aβ1–42, shortened telomere could not restore length, and then there were plenty of apoptotic cells. However, curcumin and Cur1 could bind to Aβ1–42 and antagonize neurotoxicity,thus the expression of hTERT was up-regulated, shortened telomere restored length and the numbers of cells were increased. We also observed hTERT did not increase when curcumin or Cur1 treated SK-N-SH cells without Aβ1–42 treatment. We infer that curcumin and Cur1 have no effect on hTERT up-regulation in normal cells and the treatment of them does not lead to an unlimited cell growth.

We further compared the differences in neuroprotection between curcumin and Cur1, and we found that Cur1 had a stronger protection against Aβ-induced damage than curcumin. Cur1 possesses some alterations in the chemical constitution compared with curcumin. The differences are in the substituent groups at both ends of benzene ring: the 3-methoxy of Cur1 is removed but 4-hydroxyl group remains. In our previous study, we found that 4-hydroxyl group might be responsible for the neuroprotection of curcumin and Cur1 through anti-Aβ toxicity [11].

However, while telomerase was inhibited by TERT siRNA, the neuroprotection of curcumin and Cur1 was lost. In this situation, curcumin or Cur1 no longer improved cell viability nor reduced oxidation. Meanwhile, the expression of hTERT was not up-regulated by the two compounds. So it shows that the inhibition of hTERT by siRNA is irreversible. We speculated that neuroprotective effects of curcumin and Cur1 depend on telomerase, and telomerase may be a target for therapeutic effects of curcumin and Cur1. In several studies in cancer cells, high dose of curcumin could induce telomerase inhibition, telomere shortening and apoptosis in tumour cells [35], [36]. The curcumin concentrations in these studies (25 µmol/L-50 µmol/L) were far higher than in our study (10 µmol/L); the activity of curcumin in these studies was pro-apoptotic effects on tumor cells, conversely, in our study was apoptosis inhibition. We suspect that dose-dependent relationship may explain the disparity and the effects can be reversed when curcumin is used in high enough dose; in other words, the impact on the expression of hTERT may be related to curcumin dose. Furthermore, it seems there may be cell type difference in the activities of curcumin.

In conclusion, our study indicated that Aβ could induce apoptosis, oxidative stress and inhibition of TERT expression in SK-N-SH cells. Our study demonstrated protective effects of curcumin and Cur1 against Aβ neurotoxicity in vitro. Nevertheless, the protective effects of curcumin and Cur1 were lost while telomerase was scarce. In view of the special advantages of curcumin and Cur1, such as the penetration of blood-brain barrier, we reasonably believe that curcumin and Cur1 may be a potential therapeutic agent for AD. Moreover, our data suggests that neuroprotective effects of curcumin and Cur1 depend on telomerase, and telomerase may be a target for therapeutic effects of curcumin and Cur1. These data may provide a potential therapeutic strategy for treating AD.
